# Cognitive distortions and gambling near-misses in Internet Gaming Disorder: A preliminary study

**DOI:** 10.1371/journal.pone.0191110

**Published:** 2018-01-18

**Authors:** Yin Wu, Guillaume Sescousse, Hongbo Yu, Luke Clark, Hong Li

**Affiliations:** 1 Research Center for Brain Function and Psychological Science, Shenzhen University, Shenzhen, China; 2 Shenzhen Key Laboratory of Affective and Social Cognitive Science, Shenzhen University, Shenzhen, China; 3 Donders Institute for Brain, Cognition and Behaviour, Radboud University, Nijmegen, The Netherlands; 4 Department of Experimental Psychology, University of Oxford, Oxford, United Kingdom; 5 Centre for Gambling Research at UBC, Department of Psychology, University of British Columbia, Vancouver, British Columbia, Canada; Centre for Addiction and Mental Health, CANADA

## Abstract

Increased cognitive distortions (i.e. biased processing of chance, probability and skill) are a key psychopathological process in disordered gambling. The present study investigated state and trait aspects of cognitive distortions in 22 individuals with Internet Gaming Disorder (IGD) and 22 healthy controls. Participants completed the Gambling Related Cognitions Scale as a trait measure of cognitive distortions, and played a slot machine task delivering wins, near-misses and full-misses. Ratings of pleasure (“liking”) and motivation to play (“wanting”) were taken following the different outcomes, and gambling persistence was measured after a mandatory phase. IGD was associated with elevated trait cognitive distortions, in particular skill-oriented cognitions. On the slot machine task, the IGD group showed increased “wanting” ratings compared with control participants, while the two groups did not differ regarding their “liking” of the game. The IGD group displayed increased persistence on the slot machine task. Near-miss outcomes did not elicit stronger motivation to play compared to full-miss outcomes overall, and there was no group difference on this measure. However, a near-miss position effect was observed, such that near-misses stopping before the payline were rated as more motivating than near-misses that stopped after the payline, and this differentiation was attenuated in the IGD group, suggesting possible counterfactual thinking deficits in this group. These data provide preliminary evidence for increased incentive motivation and cognitive distortions in IGD, at least in the context of a chance-based gambling environment.

## Introduction

Internet Gaming Disorder (IGD) is characterized by the failure to control one’s impulses to use online gaming [1]. Affected individuals spend many hours playing online video games such as massively multiplayer online role-playing games (MMORPGs, e.g. World of Warcraft), with apparent negative consequences upon their professional and personal life [2,3]. There is increasing interest in investigating the neurocognitive and brain mechanisms underlying IGD. For instance, IGD is associated with increased trait impulsivity [4], and cognitive impairments on tasks of impulsive choice and impulsive action [5,6]. Gaming-related stimuli increase craving among individuals with IGD, similar to the cue-reactivity response observed in substance use disorders and Gambling Disorder [7,8]. Thus, there is growing evidence suggesting that IGD shares many similarities with substance and gambling addictions, and the DSM-5 (*Diagnostic and Statistical Manual of Mental Disorders*, the fifth version) highlights IGD as a candidate behavioral addiction to be listed in the Substance-Related and Addictive Disorders category, alongside Gambling Disorder [9].

Gambling Disorder has been studied extensively in recent years as the prototypical behavioral addiction. One approach has sought to identify core cognitive distortions that occur during gambling. These distortions refer to the biased processing of chance, probability and skill in gambling behavior [10]. Two pervasive cognitive distortions are the illusion of control and the gambler’s fallacy [11]. These biases can be observed in healthy individuals, and are elevated in individuals with Gambling Disorder [12–14]. For example, using the think aloud procedure, it was shown that gamblers display high levels of erroneous verbalizations, which are associated with gambling severity [15]. Individuals with IGD may also display cognitive distortions, similar to those seen in Gambling Disorder [16]. Cognitive psychology studies have identified beliefs about game reward value and tangibility as factors contributing to persistent and excessive internet gaming [17]. Online video games also share numerous psychological ingredients ("structural characteristics") with gambling games, including audio-visual sensory feedback, fast event frequency, and the use of intermittent reward schedules [18,19], which may be linked to their excessive use [11]. Through the increased emphasis on strategy and skill in video games, gamers may develop exaggerated beliefs that their capacity to overcome and master the challenges of the game with sufficient practice, analogous to the “illusion” of control under conditions of chance. Preliminary evidence supports the notion that video game play is associated with perceptions of control, superstitious thoughts, and overestimation of skill on a gambling task [16]. However, a more systematic investigation on cognitive distortions among individuals with IGD is lacking.

The aim of this study was to investigate cognitive distortions in IGD using a trait questionnaire as well as an established laboratory gambling task designed to measure state distortions. For the trait questionnaire, we used the Gambling Related Cognition Scale (GRCS) [20] measuring the individuals’ susceptibility to distorted gambling cognitions. The GRCS has good validity in assessing individual differences in cognitive distortions, and has been found to predict impulsive choice and gambling persistence [21,22]. The GRCS includes subscales assessing beliefs in personal rituals and superstitious behaviors, beliefs about the relationship between outcomes and one’s skill or bad luck (“interpretive bias”) and beliefs about the ability to anticipate future outcomes (“predictive control”, e.g. the gambler’s fallacy). We hypothesized that the group with IGD would have elevated scores on these subscales pertaining to skill-oriented cognitions.

For the laboratory task, we administered a simulated slot machine game that was designed to elicit “near miss” outcomes [23,24]. A near-miss refers to “a special kind of failure to reach a goal, one that comes close to being successful” [25]. In real-life gambling situations, examples of near-misses would be when a slot-machine pay-line displays two cherries, with the third cherry just coming into view, or when a chosen horse finishes in second place in a neck-and-neck finish. Classically, near-miss outcomes have been associated with persistent gambling and increased motivation to play [23,26]. In games that involve genuine skills, a near-miss outcome (i.e. a shot that strikes the goalpost in soccer) might indicate that one’s skill level is high and the goal is attainable [27]. Notably, individuals with disordered gambling have been found to be more susceptible to near-miss outcomes, as reflected for instance by stronger brain responses to near-misses in the ventral striatum and midbrain, which are key nodes of the brain reward circuitry [24,28,29].

Here we administered a behavioral version of the two-reel slot machine task, with monetary reinforcement of actions leading to winning outcomes, which is known to be important in generating physiological arousal in laboratory settings [30–32]. After a mandatory phase of 30 trials, participants entered a persistence phase in order to obtain a behavioral index of gambling propensity [22,27]. Based on previous work on Gambling Disorder, we hypothesized that i) individuals with IGD would have elevated scores on the GRCS, ii) individuals with IGD would show greater persistence on the slot machine game than controls, and iii) the impact of near-misses would also be stronger in IGD than controls.

## Material and methods

### Participants

Twenty-two male participants with IGD and twenty-two healthy male controls were recruited from community using advertisements. The advertisements were posted to institutional mailing lists and online forums, targeting university students. Only males were recruited due to higher IGD prevalence in men than women [33]. Eligibility criteria for the IGD group were strictly defined. Individuals needed to meet the proposed DSM-5 diagnostic threshold for IGD (at least 5 of 9 criteria) from the international consensus group [34], as assessed based on a phone interview. In addition, included participants had to meet the following criteria as assessed from paper-and-pencil questionnaires: (1) score of 50 or more on Young’s 20-item internet addiction test (IAT) [35]; (2) online game playing time ≥ 20 hours per week. These assessments were conducted by research assistants who were psychology graduate students, and had taken a prior course in clinical psychology. They were trained and supervised on the assessment protocol (i.e. explaining the content of the questionnaires to the participant, scoring) by the first author of the paper. Among the participants who responded to the online advertisements, 57 met the DSM-5 diagnostic criteria for IGD and were invited to the lab, and 22 met the additional criteria (IAT score ≥ 50 and online playing time ≥ 20h/week). The inclusion criteria for the HCs included: (1) scores ≤ 2 in the DSM-5 diagnostic criteria for IGD; (2) Young’s IAT score < 50 (As the YIAT scale scores all internet use, some healthy control participants may get a high score due to regular use of e.g. social media, but critically the control group played video games for ≤2 hours per week); (3) online game playing time ≤ 2 hours per weeks. The Problem Gambling Severity Index (PGSI) [36] was used to screen for problem gambling; no participants met the threshold for problem gambling (score ≥ 8), and there was no group difference between the IGD (nine participants scored 0) and Controls (nine participants scored 0; see Table 1). Exclusion criteria for both groups were: history of neurological illness, previous psychiatric disorder or hospitalization, current pharmacotherapy and significant physical illness, based on participants’ self-report. At the end of the test session, participants completed two self-reported instruments: (1) the Gambling Related Cognition Scale [20]; (2) Barratt Impulsiveness Scale version 11 (BIS-11), a trait measure of impulsivity [37]. The study was conducted in accordance with the Declaration of Helsinki and was approved by the Medical Research Ethics Committee of the Shenzhen University. Written informed consent was obtained from all participants. Participants were paid 50 Chinese Yuan (~$8.72) as a flat fee. They were endowed with 30 Yuan (~$4.53) to play the gambling task. The points that were gained or lost on the task were added or subtracted from this endowment, and paid as a bonus payment (ranging from 5 [~$0.76] to 35 Yuan [~$5.29], *M* = 21.68, *SD* = 12.05, depending on the level of persistence).

**Table 1:** Demographic and behavioral characteristics of individuals with IGD and healthy control subjects. Mean (SD).

Item	IGD (N = 22)	HC (N = 22)	*t*	*Df*	*p*	Cohen’s *d*
Age	21.4 (1.30)	22.0 (1.70)	-1.20	42	.239	0.40
Year of education	14.4 (1.68)	14.4 (1.44)	-0.10	42	.924	0
DSM-5 diagnostic criteria	5.73 (0.94)	0.60 (0.85)	19.02	42	< .001	5.72
Online game playing time (hours/week)	25.00 (11.53)	0.82 (1.01)	9.80	42	.005	2.95
Young’s IAT score	60.09 (7.77)	25.32 (9.45)	13.33	42	< .001	4.02
PGSI	2.41 (2.72)	1.41 (1.65)	1.47	42	.148	0.44
BIS-11	72.64 (7.89)	65.91 (7.18)	2.96	42	.005	0.89

### Design and procedures

**Fig 1:**
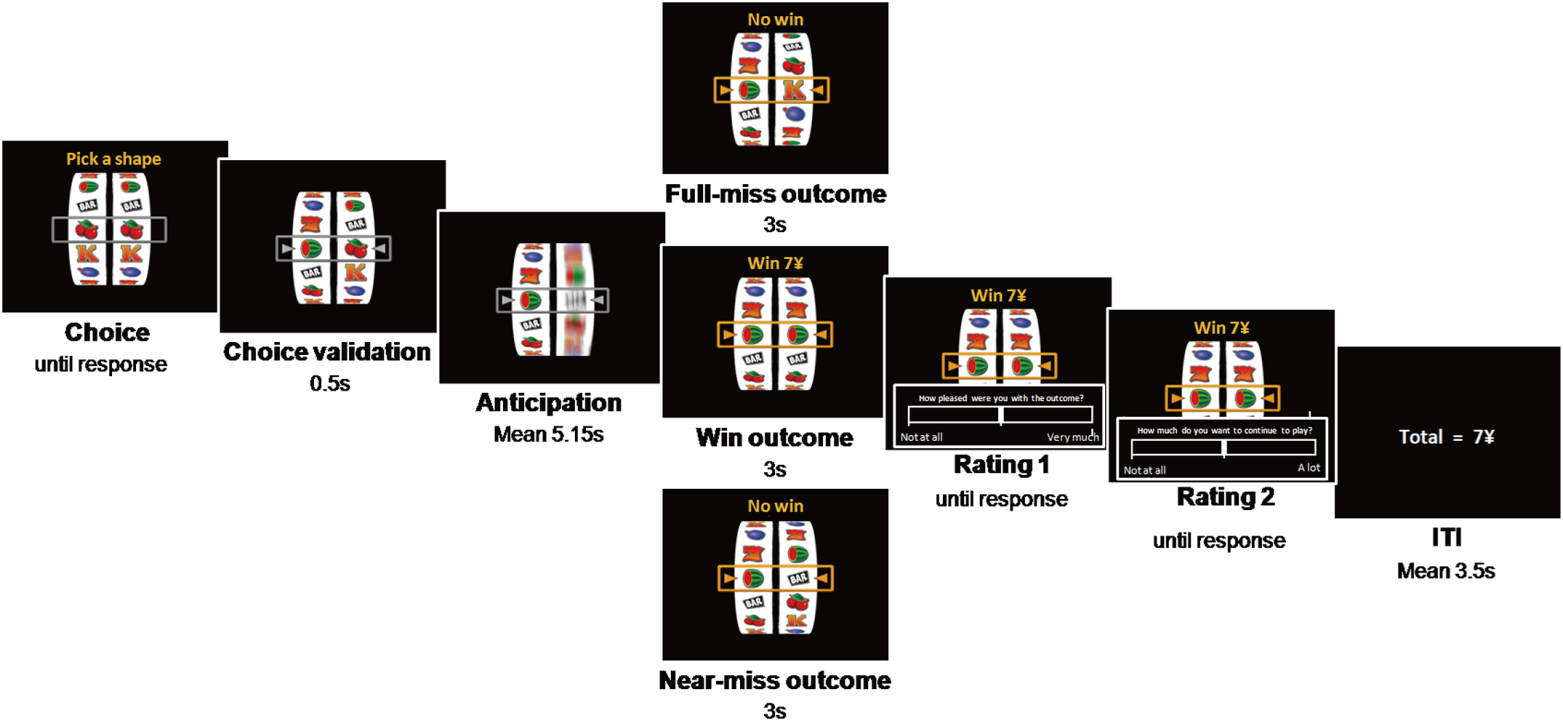
Time course of the slot machine task.

The slot machine task (see Fig 1) was adapted from our past experiment [24]. The task was programmed using Presentation Software (Neurobehavioral System Inc.), using 3D graphics and sounds to make it realistic and engaging. It involved two practice trials, followed by a fixed (“mandatory”) phase of 30 trials and then a persistence phase (maximum 30 trials) where participants could quit the task at any point. On each trial, two reels were presented that displayed the same six icons, with a horizontal payline visible centrally. Participants received 30 Chinese Yuan endowment to play with, with a fixed 1 Yuan wager on each trial. Each trial involved a selection phase where the participant chose one of the six icons on the left reel as their “play icon”, using keys to scroll the reel around and a third key to select. Following selection (no time limit), the right reel spun for an anticipation interval (3.36–6.95 seconds), during which time the reel decelerated to a standstill. In the outcome phase (3 seconds), if the right reel stopped on the chosen play icon (i.e. the icons were aligned in the payline), the participant won 7 Yuan accompanied by a cash register sound. All other outcomes were losses and were associated with a buzzer sound. Near-miss outcomes were defined as those where the right reel stopped one position away from the selected symbol. Full-miss outcomes were defined as those where the right reel stopped in one of the further positions away from the selected symbol. On each trial, two subjective ratings were taken after the outcome delivery, “How pleased were you with the outcome?” and “How much do you want to continue to play?”, both using continuous scales ranging from “not at all” to “very much”. Following the two ratings, there was a short inter-trial interval (3.5 seconds) when the cumulative earnings were displayed.

Outcomes were delivered on a pseudo-randomized sequence, such that wins occurred on one in six trials (16.7%, and thus a fair rate) and near-misses occurred on two in six trials (33.3%), with an equal number of near-misses on either side of the payline. This ensured that all participants finished the mandatory phase of the task in profit. The onset of the persistence phase was signaled by appearance of a “quit” button in the top left corner of the screen, and participants were instructed (at the beginning of the testing session) that from this point onwards, they could terminate the game at any point. No further wins were delivered during the persistence phase, which thus measures gambling under extinction [26,38]. The ratio of near-miss outcomes and full-miss outcomes was 1:2 in the persistence phase, and the subjective ratings were removed in this phase. The number of trials played in the persistence phase was taken as a measure of gambling persistence. Due to the self-paced selection of the playing symbol on each trial, the duration of the task varied (from 5.18 to 11.87 minutes (*M* = 7.67, *SD* = 1.23) for the compulsory phase, and 0 to 6.08 minutes (*M* = 2.43, *SD* = 2.15) for the persistence phase).

### Data processing and analysis

The subjective ratings data were analyzed using mixed-factors analysis of variance (ANOVA) for the different outcome types. The threshold for all statistical tests was *p* < .05 two tailed. The Greenhouse-Geisser correction for violation of the assumption of sphericity was applied where appropriate.

## Results

### Demographic and behavioral measures

The IGD and control groups did not differ reliably in terms of age, years of education, or PGSI gambling involvement (see Table 1). As expected from the eligibility criteria, the IGD group scored significantly higher on DSM-5 diagnostic criteria, YIAT scores, and online video game time per week. The IGD group showed increased trait impulsivity on the BIS-11.

### Trait measure of cognitive distortions

The IGD group had elevated Total scores on the GRCS compared to the controls. On the GRCS subscales, the difference was driven by the subscales assessing “predictive control”, “illusion of control” and “gambling expectancies” (see Table 2).

**Table 2:** Group differences on the GRCS, mean (SD).

	IGD	Controls	*t*	*df*	*p*	Cohen’s *d*
Total score						
	55.82 (21.29)	38.86 (17.50)	2.89	42	.006	0.87
Subscale						
Gambling expectancy	9.00 (4.15)	6.50 (3.33)	2.20	42	.033	0.66
Illusion of control	11.27 (5.06)	7.23 (4.97)	2.68	42	.011	0.81
Predictive control	17.36 (6.69)	11.09 (4.47)	3.66	42	.001	1.10
Inability to stop	8.59 (4.69)	6.95 (3.50)	1.31	42	.197	0.40
Interpretive bias	9.59 (5.30)	7.09 (3.60)	1.83	42	.074	0.55

### Laboratory slot machine task

#### Subjective ratings

**Fig 2:**
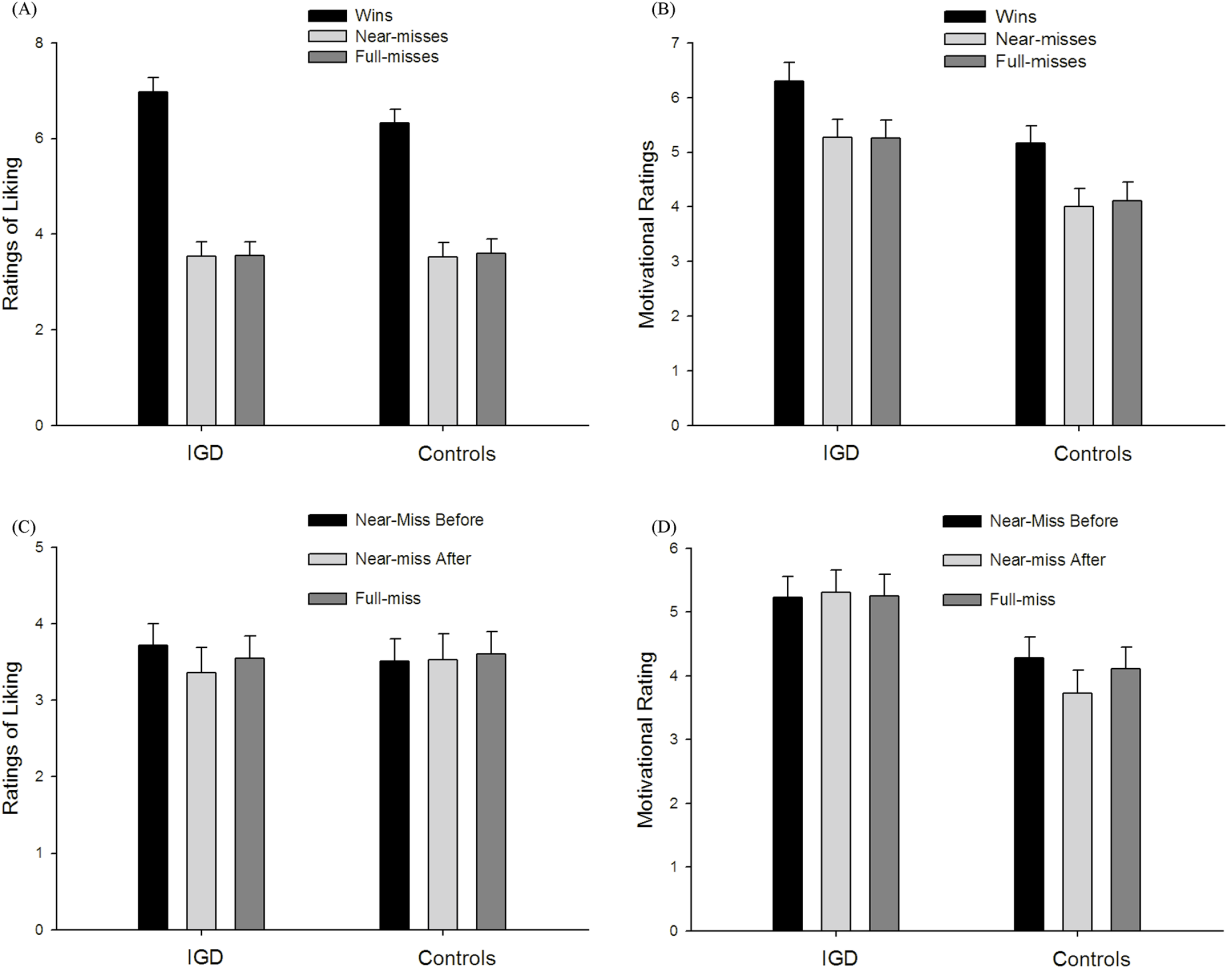
Subjective ratings in the slot machine task. (A) Liking ratings for wins, near-misses and full-misses; (B) Motivational ratings for wins, near-misses and full-misses; (C) Liking ratings for near-miss before, near-miss after and full-misses; (D) Motivational ratings for near-miss before, near-miss after and full-misses.

Ratings of ‘liking’ (“how pleased were you with the outcome”) were analyzed using a two-way mixed-factorial ANOVA with Outcome (wins, near-misses, full-misses) as a within-subjects factor and Group (IGD, controls) as a between-subjects factor. This revealed a large and significant main effect of Outcome, *F*(2, 84) = 68.05, *p* < .001, *η*_*p*_^*2*^ = .62, with higher liking ratings for wins (*M* = 6.65, *SD* = 1.43) than near-misses (*M* = 3.58, *SD* = 1.39) and full-misses (*M* = 3.53, *SD* = 1.36), both *p*s < .001 (see Fig 2A). There was no difference between near-misses and full-misses on liking ratings, *p* = .500. Neither the main effect of Group nor interaction between Group and Outcome was significant, *F*(1, 42) = .80, *p* = .375, and *F*(2, 84) = .83, *p* = .375.

In an equivalent model for motivational ratings (‘wanting’, i.e. “How much do you want to continue to play?”, see Fig 2B), there was a significant main effect of Outcome, *F*(2, 84) = 29.35, *p* < .001, _p_ η^2^ = .41, with wins (*M* = 5.74, *SD* = 1.55) being associated with a higher desire to continue gambling than full-misses (*M* = 4.68, *SD* = 1.66) and near-misses (*M* = 4.64, *SD* = 1.68), both *p*s < .001. There was no significant difference between near-misses and full-misses on motivational ratings, *p* = .61. The main effect of Group was significant, *F* (1, 42) = 7.77, *p* = .008, *η*_*p*_^*2*^ = .16, such that the IGD participants (*M* = 5.61, *SD* = 1.43) reported stronger motivations to play than the controls (*M* = 4.43, *SD* = 4.43). The interaction between Outcome and Group was not significant, *F*(2, 84) = 0.11, *p* = .904.

Thus, the IGD and control groups showed a similar response pattern on liking ratings, whereas the IGD group had higher overall motivational ratings than controls. As a direct test of this dissociation, we ran a post-hoc ANOVA model with Rating type (liking, wanting) as a further within-subjects factor, and Group (IGD, controls) as a between-participant factor. The two-way interaction between Rating type and Group was significant, *F* (1, 42) = 4.23, *p* < .046, *η*_*p*_^*2*^ = .092. Simple effects analysis confirmed our observation that the two groups did not differ on liking ratings, *t* = .90, *df* = 42, *p* = .375, but that the IGD group (*M* = 5.83, *SD* = 1.35) had higher motivational ratings than the controls (*M* = 4.81, *SD* = 0.98), *t* = 2.87, *df* = 42, *p* = .006.

In the next set of analyses, we distinguished near-misses where the reel stopped one position short of the payline (henceforth, ‘near-miss before payline’) from those where the reel passed through the payline and stopped in the next position (henceforth, ‘near-miss after payline’) [27,39]. On the liking ratings (see Fig 2C), in the 3 (Outcome: near-miss before, near-miss after, full-miss) x 2 (Group: IGD, Controls) ANOVA, neither the main effect of Outcome nor the main effect of Group was significant, *F*(2, 84) = 1.47, *p* = .237, and *F*(1, 42) = .00, *p* = .983, respectively. The interaction between Outcome and Group was not significant, *F*(2, 84) = 1.68, *p* = .192.

On the motivational ratings (see Fig 2D), the main effect of Outcome was non-significant, *F* (2, 84) = 3.06, *p* = .052, and the main effect of Group was significant, *F* (1, 42) = 6.87, *p* = .012, *η*_*p*_^*2*^ = 0.14, such that the IGD group (*M* = 5.27, *SD* = 1.52) had higher motivational ratings than the controls (*M* = 4.04, *SD* = 1.58). Notably, the interaction between Outcome and Group was significant model, *F* (2, 84) = 5.20, *p* = .007, *η*_*p*_^*2*^ = .11. The interaction was decomposed by looking at the effect of Outcome in the groups separately. In the Controls, the main effect of Outcome was significant, *F* (2, 42) = 7.28, *p* = .002, *η*_*p*_^*2*^ = .26, with motivational ratings being higher for near-miss before (*M* = 4.28, *SD* = 1.62) than near-miss after (*M* = 3.73, *SD* = 1.75), *p* = .004. In the IGD group, the main effect of Outcome was not significant, *F* (2, 42) = .16, *p* = .831. Inclusion of PGSI as a covariate in all the models reported above did not change the pattern of results.

#### Persistence behavior

By using fixed pseudo-random sequence of outcomes, all participants had the same balance of 45 Chinese Yuan upon completion of the mandatory phase of the slot machine task. The number of trials played in the persistence phase ranged from 0 to 30. The IGD group (*M* = 17.45, *SD* = 11.84) persisted longer than the controls (*M* = 9.18, *SD* = 11.01), *t* = 2.40, *df* = 42, *p* = .021, Cohen’s *d* = 0.72.

## Discussion

The present study described gambling-related cognitive distortions in a community-recruited, but rigorously defined, group with IGD, with a particular focus on their emotional responsivity to near-miss outcomes. We tested individuals with excessive use of online video games, which has become the clearest candidate phenotype in recent work on pathological gaming [34]. Past work has mostly relied on Young’s Internet Addiction Test [35] to screen participants, which quantifies the functional impact of various forms of internet use, including gaming, gambling, social media or pornography. The present study adopted the proposed DSM-5 criteria and thresholding for IGD [34], which were corroborated by high YIAT scores and at least 20 hours of online gaming per typical week. The IGD group had elevated trait impulsivity, confirming past work on IGD [40], and they had higher levels of gambling distortions on the GRCS. In the slot machine task, the IGD group played more trials in the persistence phase, and showed increased motivational ratings to play the game (‘wanting’) compared to the controls. Interestingly, the two groups did not differ on their ‘liking’ ratings for win or near-miss events. In contrast to our hypothesis, near-miss outcomes did not elicit stronger motivational ratings compared with full-misses, and there was no group difference on this measure. However, in line with our past research, the two subtypes of near-misses stopping either side of the payline had differential effects, with the near-misses before the payline increasing motivation ratings [27,39,41]. Here, we saw that this differential effect was attenuated among individuals with IGD.

The IGD group displayed higher levels of gambling distortions, showing a similar pattern at the trait level to that reported in previous work in Gambling Disorder [21]. This group difference was largely driven by increased scores on the GRCS ‘predictive control’ and ‘illusion of control’ subscales that may be considered more parsimoniously as ‘skill-oriented cognitions’ (i.e. beliefs that skills may be acquired through practice, and this could increase the likelihood of winning) [22]. In line with our observations in IGD, heightened illusion of control has been reported in regular gamblers who prefer skill-based games, such as poker and sports betting, compared with gamblers who prefer games of pure chance [42]. Previous research among gamblers has shown that video game involvement was a significant predictor of gambling-related cognitive distortions, possibly reflecting inflated confidence [16,43]. Nevertheless, in the context of gaming, there is an argument that beliefs about control might not necessarily reflect cognitive distortions, since video games involve an element of actual skill that can result in a legitimate sense of expertise and control over the game. In this regard, the increase in gambling distortions that we have observed in IGD could reflect a trait disposition or the inappropriate transfer of their extensive experience in a skilful (video gaming) environment to a chance (gambling) environment, as in the current slot machine task. This latter ‘transfer’ hypothesis could in principle be overcome with extended practice, which could be a target for future research.

In the present study, we saw a dissociation between the ratings of “how pleased were you with the outcome?” and “how much do you want to continue to play?” which may reflect underlying differences between ‘liking’ and ‘wanting’ aspects of reward processing [44]. Within this account, wanting refers to the acquisition of incentive salience to a stimulus, while liking refers to the core process of hedonic pleasure. These two systems are underpinned by distinct psychological and neural mechanisms, and can become dissociated, particularly in the context of addiction. In drug addiction, repeated consumption of addictive drugs sensitizes the mesolimbic dopamine system, the primary component of the wanting system, leading to excessive wanting for drugs and their cues. This sensitizing process is long-lasting and happens independently of the liking system, which typically remains unchanged or may develop a blunted pleasure response to the drug [45]. The incentive sensitization theory has been successfully applied to instances of behavioral addiction including Gambling Disorder in recent years [46,47]. The present data is consistent with such a mechanism in IGD. While preliminary, these findings are consistent with other evidence; for instance, cases with IGD showed an attentional bias toward computer-related cues, consistent with increased wanting and research in substance use disorders showing hyper-reactivity to addiction-related cues [48]. In the present study, the enhanced incentive motivation was also expressed as greater persistence in the extinction phase in the IGD group.

In the present study, near-miss outcomes did not elicit stronger motivation to play compared to full-misses. However, we observed a near-miss position effect such that near-misses that stop just short of the pay-line (i.e. near-miss before) primarily act to increase motivation to continue [27,39,41]. We have interpreted this position effect in terms of counterfactual thinking theory, drawing upon the distinction between additive and subtractive counterfactual thoughts [49]. Near-misses before the pay-line imply a trajectory towards the winning outcome, in which an additive counterfactual involves the mental simulation of the extra movement onto the pay-line. Notably, this position effect was attenuated among individuals with IGD, suggesting possible changes in the generation of these counterfactual thoughts in IGD. Future studies are needed to characterize more fully counterfactual processing in IGD, and indeed in other forms of addiction [50].

Some limitations should be noted. First, we used a well-established gambling procedure to probe near-miss processing in IGD, but in testing a group with gaming experience on a chance-based environment, we cannot separate a true “trait” disposition to gambling distortions from the possibility that the IGD group misapply a valid belief to an environment with which they are unfamiliar. Similarly, the lack of differentiation of the near-miss position effect in the IGD group could be interpreted as an impairment of counterfactual thinking or a more accurate perception of the neutral (non-win) outcomes in the game. Future research could separate these possibilities by testing the impact of near-misses (and other distortions) under varying levels of skill vs. chance, or by testing these effects as a function of gaming proficiency measures in IGD. Second, although none of our sample met PGSI thresholds indicative of problem gambling (≥ 8), some participants scored in the at-risk range on the PGSI, indicating gambling exposure and a low level of harm. The groups did not differ in PGSI scores and inclusion of PGSI as a covariate did not change the pattern of results. Third, psychiatric comorbidity associated with IGD was only assessed by means of self-report, without coding of severity or subclinical symptoms. Finally, the slot machine task collected subjective ratings on each trial, but these ratings imposed a short break in play that could alter the game experience, especially in the IGD group as shown previously for problem gamblers [51].

## Conclusion

Recent technological trends have seen a blurring of the lines between gaming and gambling, coming from both the “gamification” of gambling via incorporation of skill-based elements [52], and the “gamblification” of gaming via increasing use of monetization features [53]. The present study sought to explore psychological traits that are relevant to both behaviors, and such traits could be separable from traits like impulsivity that are emphasized across the addictions. The present study observed elevated levels of trait gambling distortions in IGD, in particularly in skill-oriented beliefs, highlighting the potential role of cognitive distortions in IGD. The IGD group displayed more persistent behavior in the slot machine task, consistent with increased motivation. The dissociation between ‘liking’ and ‘wanting’ during the simulated slot task is further consistent with the involvement of incentive sensitization processes in behavioral addictions.
